# Supplementary material for: Rapid turnover of life-cycle-related genes in the brown algae
Source: Genome Biol. 2019 Feb 14;20:35. doi: 10.1186/s13059-019-1630-6 (PMC6374913; doi:10.1186/s13059-019-1630-6)
Supplement: Supplementary file 3 — Protein alignments, corresponding Gblocks html file and CDS sequences for the lineage conserved gametophyte-biased genes. (ZIP 2109 kb) [file 13059_2019_1630_MOESM3_ESM.zip › Con_GA_alignments/Ec-10_006310.aa_ali.fasta-gb.htm]

Ec-10\_006310.aa.fasta.trans.aa\_ali.fasta


## Gblocks 0.91b Results

Processed file: **Ec-10\_006310.aa.fasta.trans.aa\_ali.fasta**  
Number of sequences: **2**  
Alignment assumed to be: **Protein**  
New number of positions: **1016** (selected positions are underlined in blue)

```
                         10        20        30        40        50        60
                 =========+=========+=========+=========+=========+=========+
Ec-10_006310     ------------------------------------------------MSFSVSR-YATD
MSTRG.36220.1    FSYKVFLSTPPFTTGTGTTIRCQSVFISAECCEEYAVDSPVSRCLRSCMSFSMPHGFGTD
                                                                             


                         70        80        90       100       110       120
                 =========+=========+=========+=========+=========+=========+
Ec-10_006310     DGPESLEDGLEIPGFPKPSKPEGFTGNLQGHLEKLSSNARSIHHVLETLCGGLDRLEKKM
MSTRG.36220.1    DGPESREE-MEIPGFPKAKKAEGFANSLQSHLERLSSNAQSLHHVLEALCGGMDRLEKKI
                           ##################################################


                        130       140       150       160       170       180
                 =========+=========+=========+=========+=========+=========+
Ec-10_006310     DTNSSQVEARLANIVHHISGEADEDTMSAWSSIAGGGGGGAGSPTDLGTAPSLRRRGSFG
MSTRG.36220.1    DANSAQVESRLANIVHHMSGEADEDTMSAWGSLG----GGLASPAELGPSLPLRRRSSFG
                 ################################      ######################


                        190       200       210       220       230       240
                 =========+=========+=========+=========+=========+=========+
Ec-10_006310     MQMASCSEDSPFDFRNDKISLDEELANVGRDGDESEDVVTSFGGVVERAKDVALQ--NEG
MSTRG.36220.1    MHMSSFSEEPFLEPPVGRTSLNGEGADVARSGDQGENDLGTFGSVVDRTKEKATGGGTEA
                 ########           ##################################       


                        250       260       270       280       290       300
                 =========+=========+=========+=========+=========+=========+
Ec-10_006310     GGRGEVERAAILTAPGNSSSAQATLKPTVVAAGPSASETLEN---TDEHQNPSEAKDLSS
MSTRG.36220.1    QTAGQSDLSSSDRNVAGSFIGAKNHGPAVVATTPKKEEDQGRGRMVPEEPNNAATRRPAS
                                  #####################         #############


                        310       320       330       340       350       360
                 =========+=========+=========+=========+=========+=========+
Ec-10_006310     ISSPTDTGSPREEDRELPTGLVSGGEDTND--------------LHPQRRRSNQDDQSDG
MSTRG.36220.1    ETAEERPAVSPAVDRKGSDGVSSNPPNSGDGRAKKALASPEGIKVLQPRRRSNQDDQSDG
                              #################                  ############


                        370       380       390       400       410       420
                 =========+=========+=========+=========+=========+=========+
Ec-10_006310     SNSKLNSARAEEGRNDVMVVSKQESSVGGESVLGEEAEVPGEGNDFVKTAFSPQASIDSG
MSTRG.36220.1    LNSKINSARSGEDERDPRTSMSEID-----SVHGEETASNAEGSEYPLTALIVEGSVESG
                 ################              ##############################


                        430       440       450       460       470       480
                 =========+=========+=========+=========+=========+=========+
Ec-10_006310     VLDSRLIARQRWIWAFGRICQLVRRRKRKQFEIMSQRATDRTVRMGSRVSVVEKRLDETT
MSTRG.36220.1    GSDARYIARQRWIWAFGKVCQLVRRRKRKQFEIMSQRATDRTVRMGSRVSVVEKRLDETR
                 ############################################################


                        490       500       510       520       530       540
                 =========+=========+=========+=========+=========+=========+
Ec-10_006310     EHLLVVNQLRDKSGSHTAQIKNLNEMVKELCKLLGMEEKFNGIVHATKKMVEEKFKSDMT
MSTRG.36220.1    EHLLVVDRLKDKSSAHTVQIKNLGETVTSLCKLFGVEERLRTTMQDSQEKLETSLKSDLS
                 ######################################             #########


                        550       560       570       580       590       600
                 =========+=========+=========+=========+=========+=========+
Ec-10_006310     ELETRLASGHRELETSLESEIRRNGTKLEDMDDRYARSEEALRRAEERIGEVQEEMHAVG
MSTRG.36220.1    DLEAKLTSRQYDMEKSLDSDIQRHGSKLEDIDDRYARSEDALRRAEERIGEVQEEISGVT
                 ############################################################


                        610       620       630       640       650       660
                 =========+=========+=========+=========+=========+=========+
Ec-10_006310     ESISDVERRLRVDEDRLGSLENLKKAHQRRLSELRVQLESGTPAAGGAVPIETEPEETDA
MSTRG.36220.1    QSISEIERQLRVDQGRLDTLENLKKAHQRRLSELRVKLEDSTTSAGIAVPIDTEPVETDD
                 ############################################################


                        670       680       690       700       710       720
                 =========+=========+=========+=========+=========+=========+
Ec-10_006310     ERMARLLKTAGTATEFLAEDLDKILDSNGGNAGDATATAADNAEANHELGQVLSARSSAS
MSTRG.36220.1    ERMNRHLRAAGAAAEFLSTDLGKILDNSSRSSSDLSCAALTATNDSAAKNDSLRDAGVHN
                 #######################################                     


                        730       740       750       760       770       780
                 =========+=========+=========+=========+=========+=========+
Ec-10_006310     N--VDHALQELRHVSVNAVECSRLCNDVASASGQAERDEIGPDGGGAGSDSNVDAAVRRV
MSTRG.36220.1    DKDGGHIMERLRHISIEAAEYSSLCSNS--------PNEKGD------DDLSIRTAVERA
                      ####################                        ###########


                        790       800       810       820       830       840
                 =========+=========+=========+=========+=========+=========+
Ec-10_006310     EALCGHIQDLLSTQ-----DKNISSSKNKAELSA--TGEIERQGSLEGNGGVGLK-----
MSTRG.36220.1    EALCGRIDALLSTPKGSGHNNNVDNGDDGMEIAAPRTTALARNGSSDSNSDPGHKRSANS
                 #############        #############  ###################     


                        850       860       870       880       890       900
                 =========+=========+=========+=========+=========+=========+
Ec-10_006310     ----IDDIVCPNGRKDLVQRLTAARDALLPVIGERSSVGSLRVAFLGLTASVRANLEEKQ
MSTRG.36220.1    NVSSIADVVCPNGRKDLALRLAAARDAFLPLIGECTNLGSLRMDLLRLTTSVGISLEPKQ
                     ########################################################


                        910       920       930       940       950       960
                 =========+=========+=========+=========+=========+=========+
Ec-10_006310     ARDLMEERWNDLRGRVSGLDTSVSDIKDSLHDEGHHRYG-SLDMDSD----N-----GED
MSTRG.36220.1    GLDTLEARWKTVSSHVSGLADSVNDIESSLHSNRQHRRGSTLDDDGGGGGDGVGGVTEED
                 #######################################                   ##


                        970       980       990      1000      1010      1020
                 =========+=========+=========+=========+=========+=========+
Ec-10_006310     LEERLGCKADVSWVQRELQRLWDALNARAMAAVGAMSPRTGAGVTEGHGGDPSRPRSAPP
MSTRG.36220.1    LDDRLGYKADVSWVQRELKRLWEALDSRAMASVVAAS----AAATAGQDGDASRPRSAPS
                 #####################################    ################## 


                       1030      1040      1050      1060      1070      1080
                 =========+=========+=========+=========+=========+=========+
Ec-10_006310     TN-------YDKENHNPETAGQEEGDLDASPSQ-ANSRVISGSLPSSALSTALGGGGGGG
MSTRG.36220.1    SKDRSSSASNNKENNNPETAGEEESDVDTSVSLTPNSRAISTSLPSSSS----------P
                            #####################   ############             


                       1090      1100      1110      1120      1130      1140
                 =========+=========+=========+=========+=========+=========+
Ec-10_006310     GGGSQGAVGGRRASFNEGASLIKDLMKKTSRLEQQMATKADNEEVMKALASVGNKLRKVG
MSTRG.36220.1    SSGSLQAEHRSRSSFNEGSSLIKDLLRKTSRLEQQMSSKADSDEVMKTLSSVGNKLRKVG
                   ##########################################################


                       1150      1160      1170      1180      1190      1200
                 =========+=========+=========+=========+=========+=========+
Ec-10_006310     IVIPAMQEELANKIERKDLAKLVSMVSNGGGTGEPPPG----GHEGPHNNGDGSAVVASR
MSTRG.36220.1    VVIPAIQEELSQKIERKDLAKLVALVSSGGNLSDAPPGEYGSGKNPSDDKNDTTAVLASR
                 ######################################    ##################


                       1210      1220      1230      1240      1250      1260
                 =========+=========+=========+=========+=========+=========+
Ec-10_006310     LNSKFRCLSCDRPLPALGPPGPPKLGASGLSMAGAGTPRSPQKTSS---QQRLKLTAAEE
MSTRG.36220.1    LNSKFRCLSCDRPLPALGPPGPPKLNASGLSIAGAGAPRSPQRTKASPGQHRVRQTAGG-
                 ############################################                


                       1270      1280      1290      1300      1310      1320
                 =========+=========+=========+=========+=========+=========+
Ec-10_006310     PGADKNSRPGSPATTSPAVEDVGDVAGWGRGS---SNMMIPGGASDMESAYGNGGKDAGA
MSTRG.36220.1    SGGDASSRPNSPAVVSPTLEDVGDVAGWGSAGGEGSGMTLP-RASDSDSACGHSKDSAGS
                  ############################              #################


                       1330      1340      1350      1360      1370      1380
                 =========+=========+=========+=========+=========+=========+
Ec-10_006310     RRRQKQQHRQHHEMDQPPGRLEPLGANGEHMAGVLSRYPRMMPPPSRIRTAAGGGIGSRP
MSTRG.36220.1    RWRHQQQMHQYRGGEPPGGRLEPLGANGEQM-----------------------------
                 ###############################                             


                 
                 ========
Ec-10_006310     SSSSGGRI
MSTRG.36220.1    --------
```

```
Parameters used
Minimum Number Of Sequences For A Conserved Position: 2
Minimum Number Of Sequences For A Flanking Position: 2
Maximum Number Of Contiguous Nonconserved Positions: 8
Minimum Length Of A Block: 10
Allowed Gap Positions: None
Use Similarity Matrices: Yes
```

```
Flank positions of the 22 selected block(s)
Flanks: [71  152]  [159  188]  [200  233]  [258  278]  [288  300]  [314  330]  [349  376]  [391  518]  [532  699]  [726  745]  [770  793]  [802  814]  [817  835]  [845  939]  [959  997]  [1002  1019]  [1032  1052]  [1056  1067]  [1083  1178]  [1183  1244]  [1262  1289]  [1304  1351]  

New number of positions in Ec-10_006310.aa.fasta.trans.aa_ali.fasta-gb:  1016  (73% of the original 1388 positions)
```
